# Bismuth Sulfide Nanoflowers for Detection of X-rays in the Mammographic Energy Range

**DOI:** 10.1038/srep09440

**Published:** 2015-03-24

**Authors:** Shruti Nambiar, Ernest K. Osei, John T. W. Yeow

**Affiliations:** 1 Department of Systems Design Engineering, 200 University Avenue West, University of Waterloo, Waterloo, Ontario, Canada, N2L 3G1; 2 Waterloo Institute for Nanotechnology, 200 University Avenue West, University of Waterloo, Waterloo, Ontario, Canada, N2L 3G1; 3 Department of Medical Physics, Grand River Regional Cancer Centre, Kitchener, Ontario, Canada, N2G 1G3; 4 Department of Physics and Astronomy, 200 University Avenue West, University of Waterloo, Waterloo, Ontario, Canada, N2L 3G1

## Abstract

The increased use of diagnostic x-rays, especially in the field of medical radiology, has necessitated a significant demand for high resolution, real-time radiation detectors. In this regard, the photoresponse of bismuth sulfide (Bi_2_S_3_), an n-type semiconducting metal chalcogenide, to low energy x-rays has been investigated in this study. In recent years, several types of nanomaterials of Bi_2_S_3_ have been widely studied for optoelectronic and thermoelectric applications. However, photoresponse of Bi_2_S_3_ nanomaterials for dosimetric applications has not yet been reported. The photosensitivity of Bi_2_S_3_ with nanoscale “flower-like” structures was characterized under x-ray tube-potentials typically used in mammographic procedures. Both dark current and photocurrent were measured under varying x-ray doses, field sizes, and bias voltages for each of the tube potentials – 20, 23, 26 and 30 kV. Results show that the Bi_2_S_3_ nanoflowers instantaneously responded to even minor changes in the dose delivered. The photoresponse was found to be relatively high (few nA) at bias voltage as low as +1 V, and fairly repeatable for both short and long exposures to mammographic x-rays with minimal or no loss in sensitivity. The overall dose-sensitivity of the Bi_2_S_3_ nanoflowers was found to be similar to that of a micro-ionization chamber.

One dimensional (1-D) nanostructures of metal chalcogenides, especially those of Bi_2_S_3_, are among the most widely studied. Bi_2_S_3_ is an n-type crystalline semiconductor with direct bandgap in the range of 1.3–1.7 eV^1^,^2^,^3^ Several studies have reported interesting morphologies of Bi_2_S_3_ in the form of nanoparticles, nanorods, nanotubes, nanowires, nanoflakes and nanoflowers^1^,^4^,^5^,^6^,^7^,^8^,^9^. These 1-D nanostructures of Bi_2_S_3_ have been reported to exhibit enhanced electrical, thermal and optoelectronic properties and thereby are extensively investigated for a variety of applications such as photovoltaics, thermoelectrics, infrared spectroscopy, and field emission^8^,^9^. In a recent study, polymer-coated Bi_2_S_3_ nanoparticles were used for in vivo x-ray imaging applications as contrast agents in x-ray computed tomography^10^. The nanoparticles showed significantly higher x-ray absorption (five-folds) in comparison to the conventional iodinated contrast agents. The relatively high effective atomic-number (Z) of Bi_2_S_3_ allows it to undergo photoelectric interaction with a wide range of x-ray energies, making it a suitable material for clinical x-ray dosimetry. Subsequently, there is a growing interest to use bismuth-based materials for high-energy dosimetric applications.

Our group has previously reported x-ray photoconductivity measurements from thin films of carbon nanotubes (CNT), and quantum dots (zinc oxide and cadmium telluride) for direct detection of therapeutic x-rays^11^,^12^,^13^,^14^. Since the atomic coefficient (i.e. the interaction cross-section) dependence for photoelectric absorption is directly proportional to Z^4^^15^, nanomaterials with high Z are most likely to generate detectable charge carriers at very low doses or low-energy x-rays, improving the sensitivity of the dosimeter. Lobez and Swager reported resistivity-based detection of gamma rays using multi-walled CNTs coated with poly (olefin sulfone) (POS) doped with bismuth. Due to its high Z, bismuth was chosen as a dopant to increase the interaction cross-section of the low Z composite of POS/CNT^16^. Similarly, Zhong et al. reported composites of bismuth iodide particles and conjugated polymers to detect gamma rays based on changes in the photoluminescence intensities of the composites upon irradiation^17^. Others have incorporated bismuth oxide nanoparticles into organic semiconducting polymers to increase the absorption cross-section, and thereby the sensitivity, for applications in large-area, x-ray dosimetry^18^,^19^. High Z nanoscale materials can occupy more volume fraction of the active detection region at relatively low weight percentage in comparison to the bulk. Moreover, both experimental and simulation studies on different types of nanocrystalline materials exposed to various sources of radiation showed enhanced resistance to radiation-induced material degradation^20^,^21^,^22^,^23^,^24^,^25^. Furthermore, some studies have also reported increased x-ray interactions (i.e. attenuation properties) of nanocomposites in comparison to those of the microcomposites, both irradiated with mammographic x-ray energies^26^,^27^,^28^. High energy photoresponse of high Z nanomaterials such as Bi_2_S_3_, therefore, has huge technological importance for the development of efficient and durable dosimeters.

In the context of synthesis of Bi_2_S_3_ nanostructures, solvothermal or hydrothermal method is one of the most commonly used solution-based processes. The hydrothermal technique allows control over the morphology by varying reaction parameters such as reaction time, temperature, precursors, and soft templates (surfactants, complexing agents, biomolecules^9^,^29^ or polymers)^30^. In this study, Bi_2_S_3_ nanostructures were synthesized through the hydrothermal process using polyethylene glycol (PEG) as a template to obtain flower-like nanostructures. To the best of our knowledge, this is the first study to report the x-ray photoresponse of the nanoflower-like structures of Bi_2_S_3_. The photoelectric response of micron-sized Bi_2_S_3_ units of “flowers” with “petals” of nanorods were measured under low x-ray energies, 20 to 30 kV, typically used in mammographic tomosynthesis. The nanoflowers were found to be extremely sensitive to the low x-ray energies at bias voltages as low as 0.2 V. The photocurrents, at 30 kV, were found to be ~3 times higher than the dark current values at all three bias voltages employed in this study. The photoresponse curves were stable and repeatable at different doses, exposure time and field sizes for all of the x-ray tube-potentials (20 to 30 kV). High sensitivity to low-energy x-rays along with other results suggests that the flower-like nanostructure of Bi_2_S_3_ is a potential material for use in semiconductor-based x-ray detection systems.

## Results

**Figure 1:**
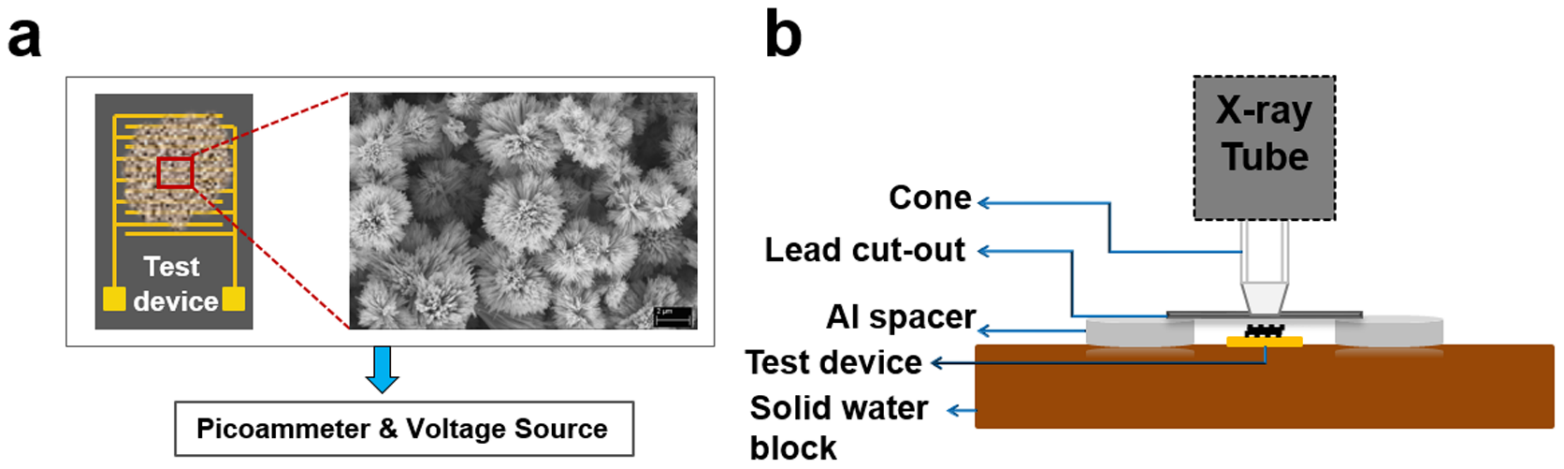
Schematic of the experimental setup. (a) The Au/Cr IDE device with Bi_2_S_3_ nanoflowers was connected to a picoammeter (also a voltage source) which allowed digitized data acquisition of the current measurements under a given bias voltage. The morphology and the overall unit size of the nanoflowers, in the range of 3 to 5 μm, can be observed in the field emission scanning electron microscopic image. (b) The Au/Cr IDE device was placed on a solid water block to allow backscattering of incident x-rays. In order to minimize radiation-induced effects on areas other than the active region of the IDE device, such as the contact pads, by placing a lead sheet with a cut-out of desired field size on Al spacers on either sides of the device. The x-ray source was positioned normal to the IDE device and the non-shielded region of the device was directly exposed to x-rays of different energies determined by the tube potential of the source.

In order to measure the x-ray induced photocurrent, interdigitated electrodes (IDE) of gold/chrome (Au/Cr) were patterned on silicon-nitride-coated silicon (Si) wafer using photolithography. Each IDE had an area of 1 cm^2^ with an electrode width and spacing of 50 μm respectively. A film of Bi_2_S_3_ nanoflowers was deposited on the IDE (Au/Cr/SiNx/Si) using dropcast method. The average unit size of the hydrothermally synthesized Bi_2_S_3_ nanoflowers was found to be ~4 μm consisting of nanorods with diameters of ~100 nm (Figure 1a). The x-ray field size was determined with a lead cut-out of 1 cm diameter placed on top of 4 mm thick spacers such that the cut-out exposed only the active area of the IDE to a 1 cm diameter cone attached to the head of the x-ray tube (Figure 1b). The photocurrents of the device with and without Bi_2_S_3_ nanoflowers in response to changes in x-ray beam intensity were measured under (i) x-ray tube potentials in the mammographic range: 20, 23, 26, and 30 kV, (ii) Four different doses at each of the tube potentials. (iii) Three operating bias voltages: +0.2, +0.4 and +1 V, and (iv) Four different field sizes (0.4, 0.6, 0.8 and 1 cm diameter) for two peak voltages (20 and 30 kV).

**Figure 2:**
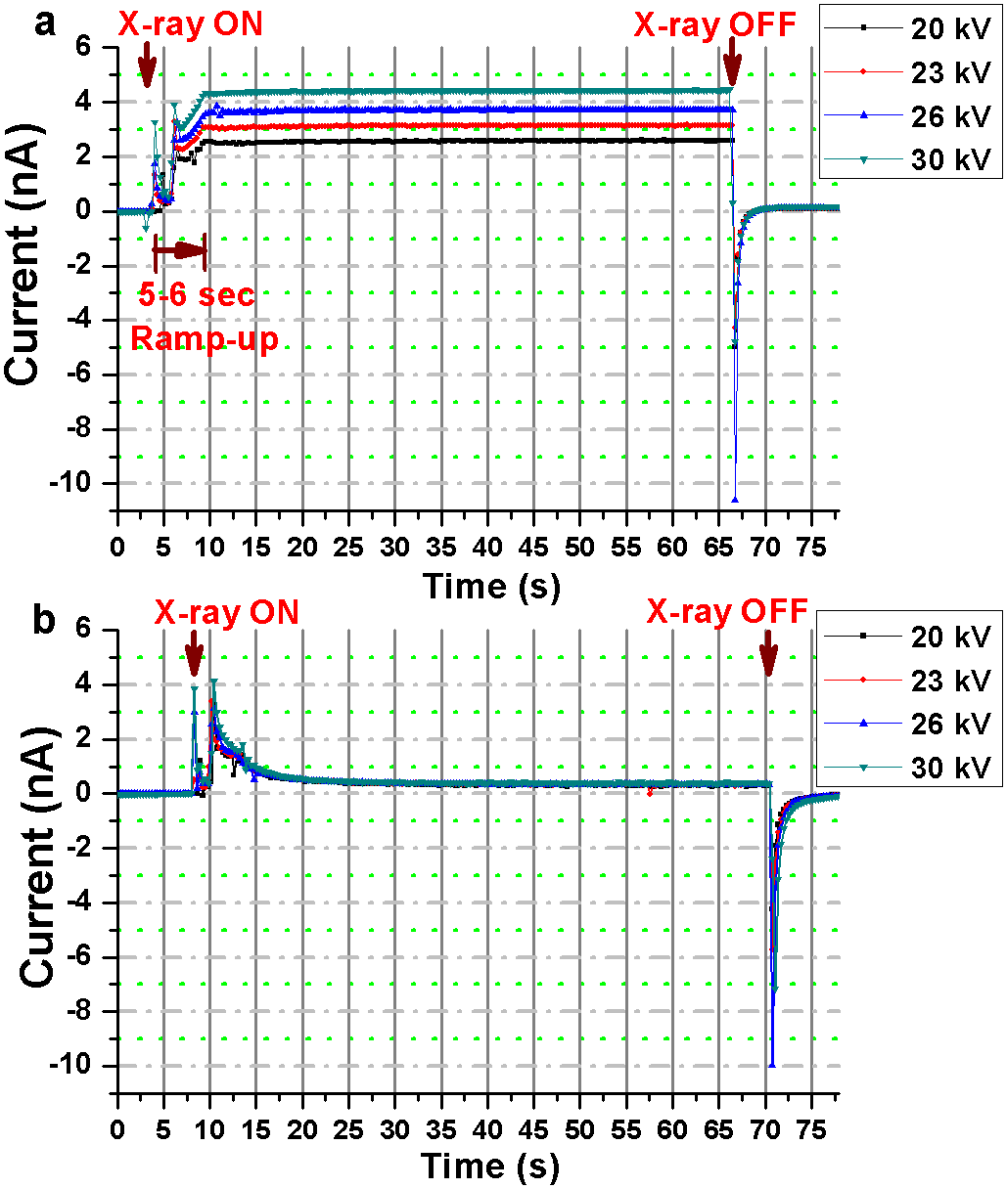
X-ray induced current in the IDE device with (a) Bi_2_S_3_ nanoflowers, and (b) in the substrate (reference device). The IDE devices were operated under a bias voltage of +1 V at four different x-ray tube-potentials of 20, 23, 26 and 30 kV, each set to a tube current of 20 mA.

**Figure 3:**
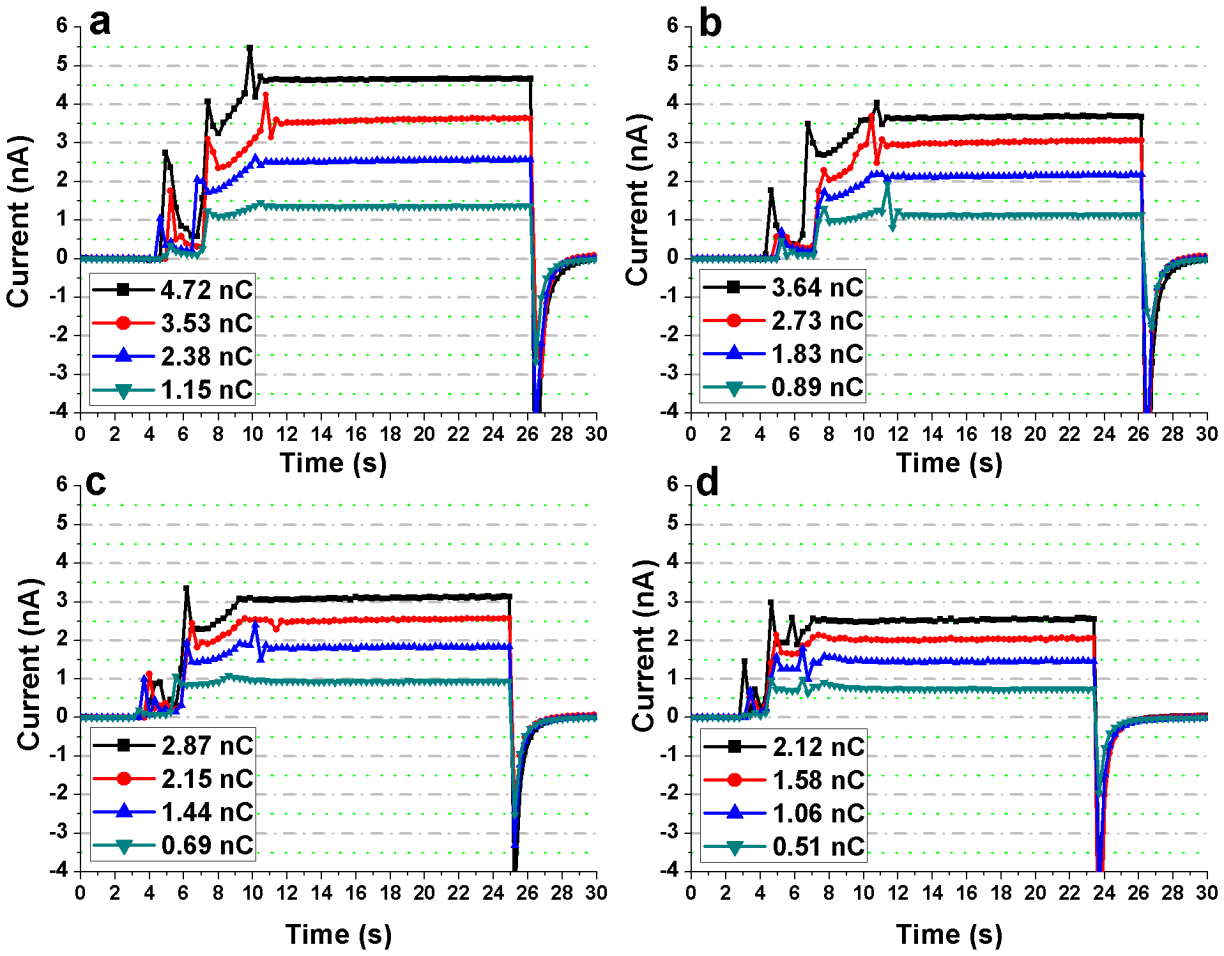
Photoresponse of the Bi_2_S_3_ nanoflowers to four different x-ray doses at a tube potential of (a) 30 kV, (b) 26 kV, (c) 23 kV, and (d) 20 kV. For each tube potential, the electrometer readout (measurements from the micro-ionization chamber) corresponding to the four different x-ray doses (D-1, D-3/4, D-1/2, and D-1/4) are shown in the legend. The exposure time was set to 18 s for all measurements.

**Figure 4:**
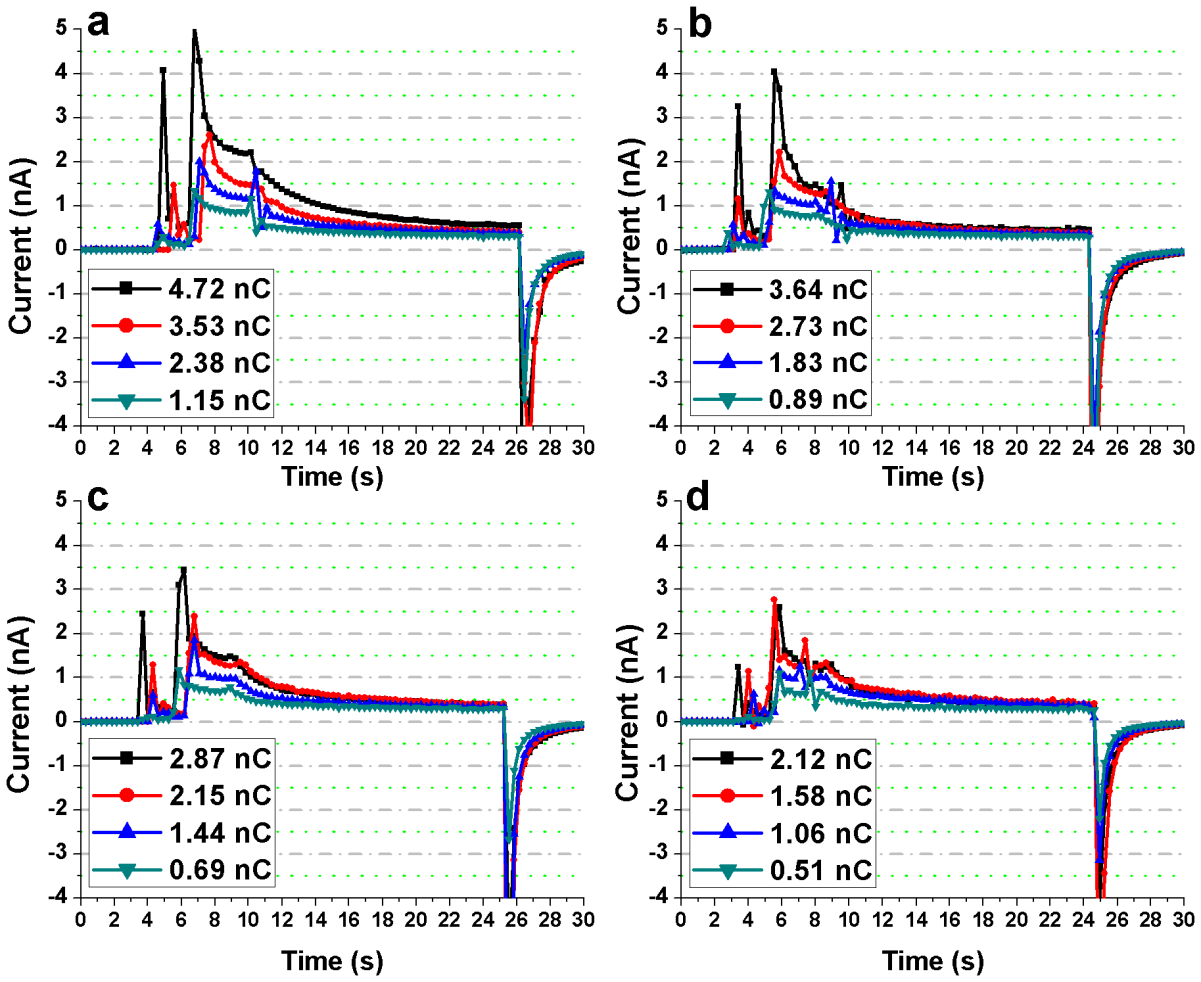
Photoresponse of the substrate (reference device) to four different x-ray doses at a tube potential of (a) 30 kV, (b) 26 kV, (c) 23 kV, and (d) 20 kV. For each tube potential, the electrometer readout (measurements from the micro-ionization chamber) corresponding to the four different x-ray doses (D-1, D-3/4, D-1/2, and D-1/4) are shown in the legend. The exposure time was set to 18 s for all measurements.

**Figure 5:**
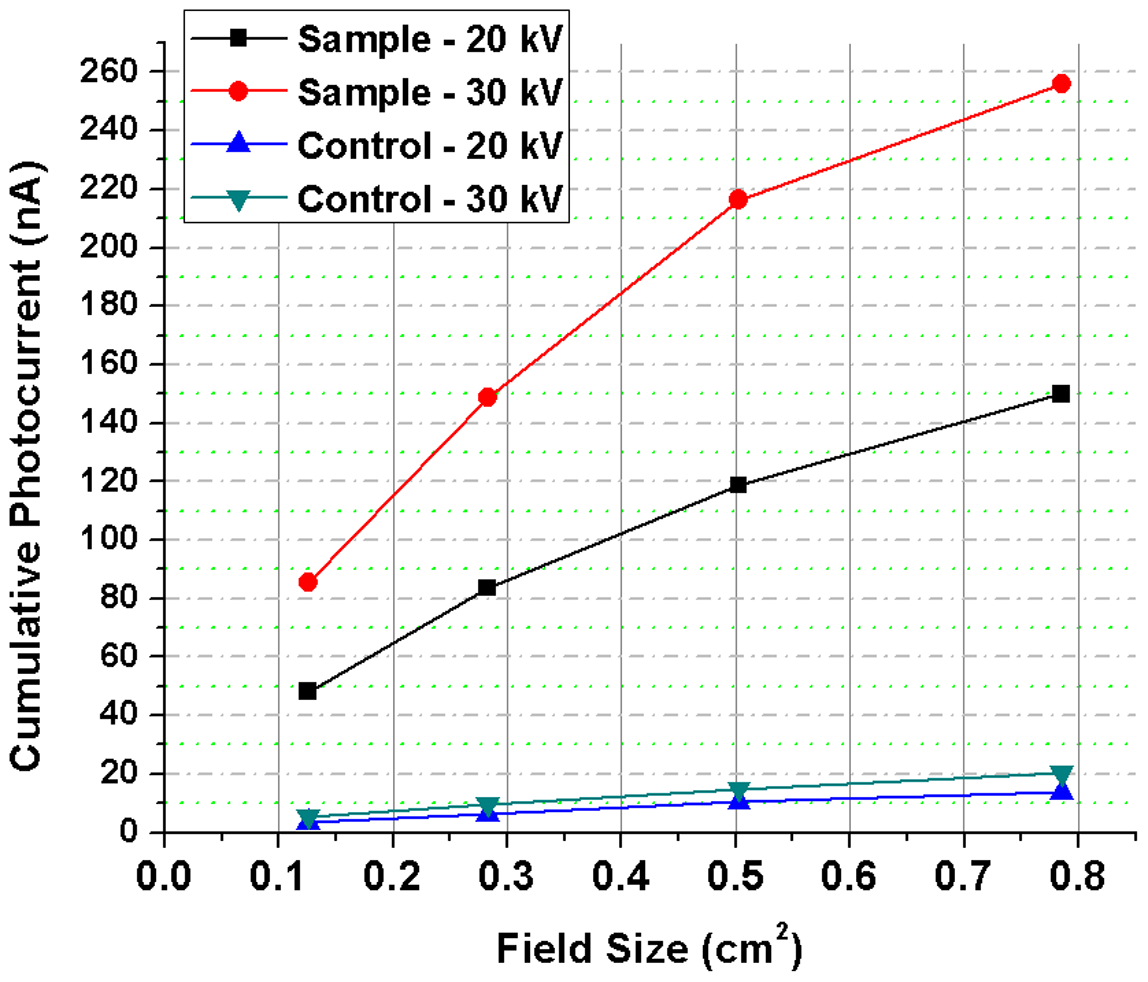
Cumulative photocurrents as a function of x-ray field size (0.4, 0.6, 0.8 and 1 cm diameter). The cumulative response at each of the field sizes was obtained by adding the instantaneous measurements for a fixed time interval for both the sample (Bi_2_S_3_ nanoflowers) and the control (reference device) at the x-ray tube potentials of 20 and 30 kV.

**Figure 6:**
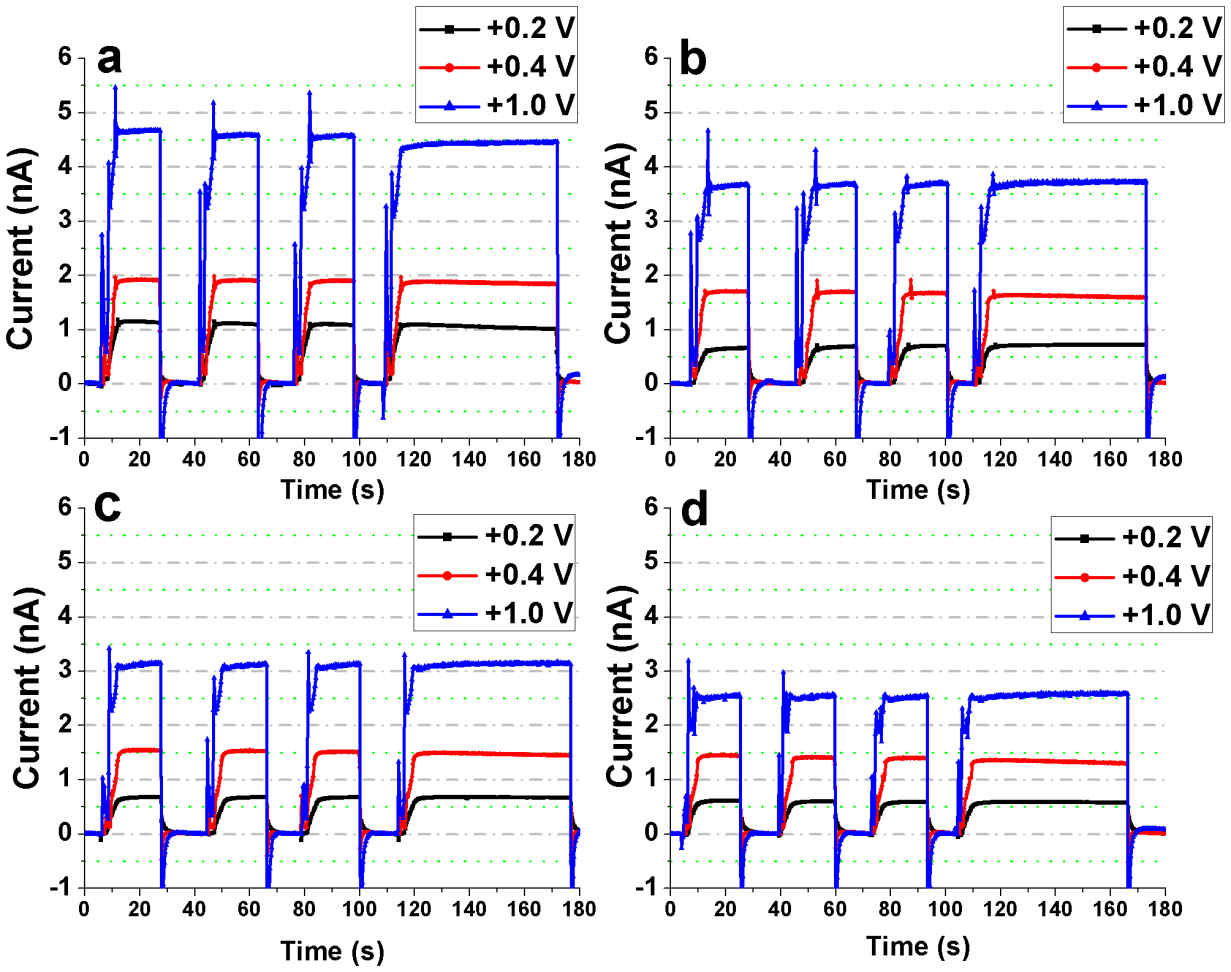
Assessment of repeatability of the measurements obtained from the Bi_2_S_3_ nanoflowers. The IDE device with the Bi_2_S_3_ nanoflowers was exposed to (a) 30 kV, (b) 26 kV, (c) 23 kV, and (d) 20 kV x-rays. At each of the tube potentials, the device measurements were recorded for three short-term (18 s) x-ray exposures followed by a long term (1 min) exposure. This was repeated for each of the three bias voltages – +0.2, +0.4 and +1 V.

**Figure 7:**
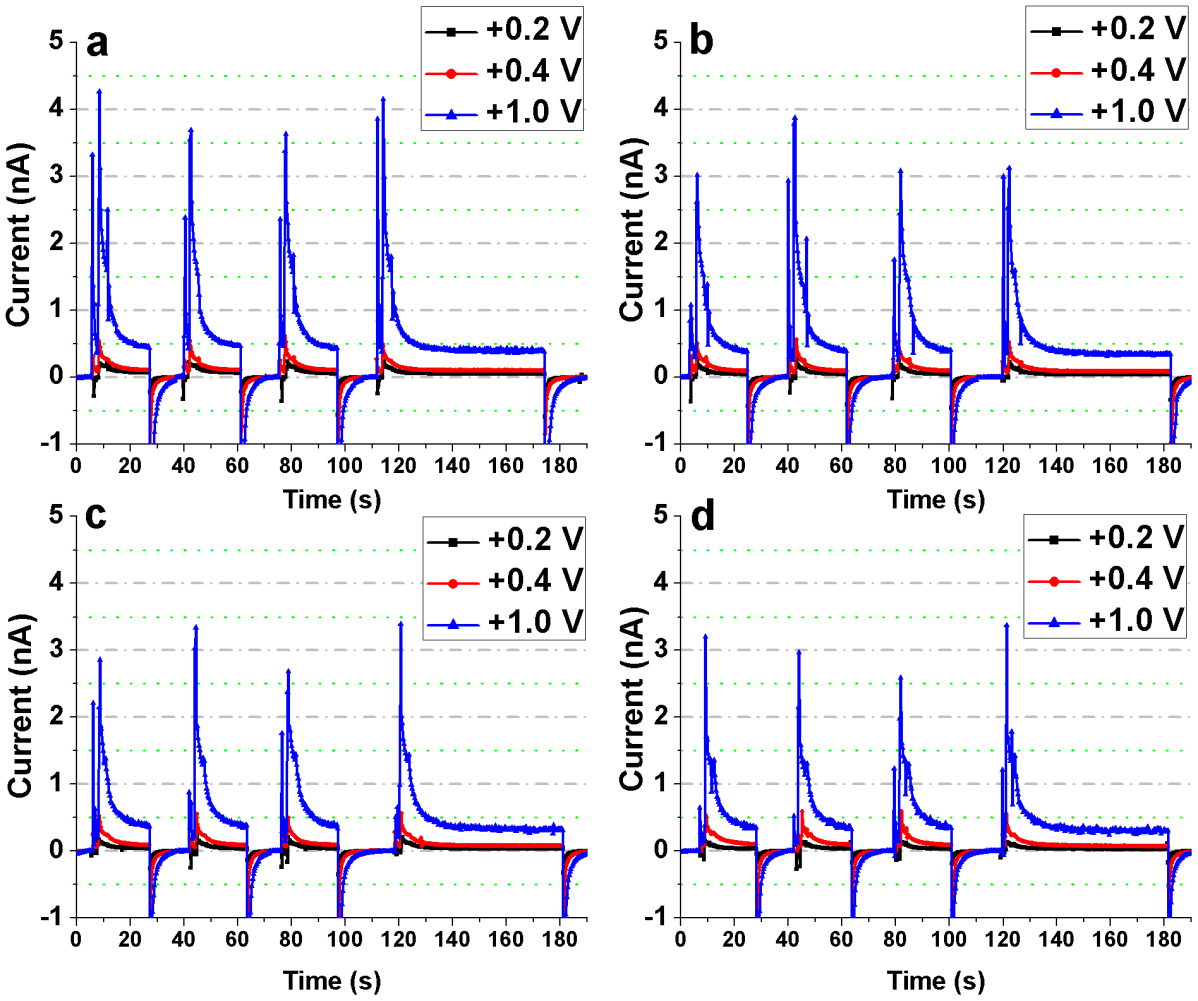
Assessment of repeatability of the measurements obtained from the substrate (reference device). The IDE device (without the Bi_2_S_3_ nanoflowers) was exposed to (a) 30 kV, (b) 26 kV, (c) 23 kV, and (d) 20 kV x-rays. At each of the tube potentials, the device measurements were recorded for three short-term (18 s) x-ray exposures followed by a long term (1 min) exposure. This was repeated for each of the three bias voltages – +0.2, +0.4 and +1 V.

**Figure 8:**
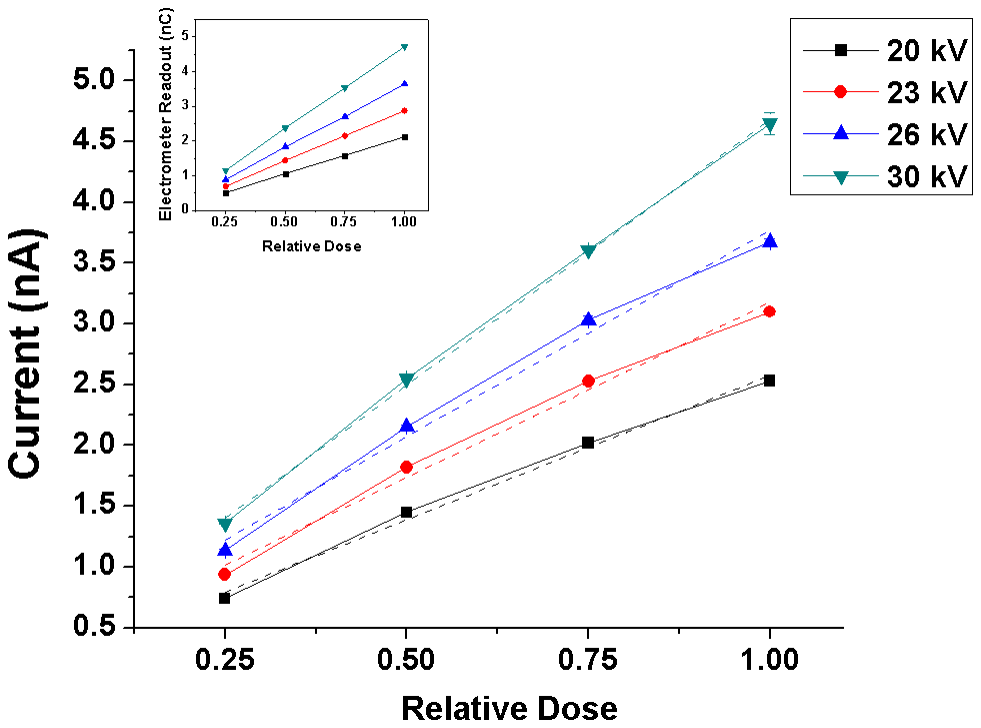
X-ray sensitivity curves of the Bi_2_S_3_ nanoflowers at tube-potentials - 20 to 30 kV. A linear fit is shown as dotted lines. The micro-ionization chamber readings for all tube potentials are shown in the inset for comparison with the photoresponse obtained from the Bi_2_S_3_ nanoflowers.

For a given bias voltage, leakage (or dark) currents were recorded for both the Bi_2_S_3_ (sample) and the reference (substrate) devices. All measurements were conducted after 15 min required for stabilization of the transient dark current, and an average of the dark currents recorded just before each exposure, was subtracted from all the measurements to obtain the data presented in Figures 2, 3, 4, 5, 6, 7, 8. For all measurements, a ramping-up period of 5–6 s was observed for the x-ray tube to attain the preset tube potential (kV) and intensity (mA). The ramp-up time was also confirmed from ionization chamber (Farmer-type chamber) measurements for all the x-ray energies used in this study.

### Effects of tube potential

Figures 2a and 2b show the effects of x-ray tube potential on the x-ray induced photocurrents for the device with and without Bi_2_S_3_ nanoflowers, respectively, when exposed to x-rays for a duration of 1 min under a bias voltage of +1 V. All the measurements were performed such that the x-ray cone was in contact with the lead cut-out used to set the field size. The initial ramp-up fluctuation was observed for both the devices (with and without the Bi_2_S_3_ nanoflowers), followed by a very stable photocurrent. The measurements from the Bi_2_S_3_ device were found to be about 5, 6, 7 and 9 times the photoresponses obtained from the reference device at the ‘x-ray ON’ state for tube-potentials 20, 23, 26 and 30 kV respectively. For all tube potentials, the reference device showed a rapid loss of charge-carriers for duration of about 18 s, followed by a plateau of steady photoresponse. In contrast, the photoresponses from the Bi_2_S_3_ device were found to be fairly stable after the initial fluctuation during the ramp-up. A “negative” current or reversal in the current flow, at the instance of ‘x-ray OFF’ state, was observed for all measurements which may be attributed to the charge trapping and release mechanism(s) from the substrate; also evident from the similar behaviour observed in the reference device at ‘x-ray OFF’ states (Figure 2b). In order to identify the x-ray interaction processes responsible for the relatively high photocurrents in the Bi_2_S_3_ nanoflowers, energy-weighted effective Z of Bi_2_S_3_ was determined using Auto-Zeff simulation software^31^ with inputs from an x-ray spectra calculation program known as SpekCalc^32^,^33^. The SpekCalc results for the mean energy of the x-ray tube potentials 20, 23, 26 and 30 kV were 9.78, 10.6, 11.4, and 12.4 keV respectively (see spectra for each of the tube potentials in the supplementary Fig. S4 online). The energy-weighted effective Z of Bi_2_S_3_ for monoenergetic beams of 10 and 13.42 keV, calculated using the Auto-Zeff software, was found to be 46.49 and 45.06 respectively (see supplementary Fig. S6 online). Furthermore, the atomic interaction of x-ray energies <12 keV with an effective Z within the range of 45 to 47 is predominantly photoelectric effect^15^. Based on the incident energy and the thickness of the dropcasted film of Bi_2_S_3_ nanoflowers exposed to x-rays, the photoelectric effect should allow partial or complete absorption of an incident photon through transfer of energy, typically, by knocking off a core shell electron from Bi_2_S_3_. The kinetic energy of the knocked off electron is equal to the difference between the incident beam energy and the binding energy of the electron in the core shell. The energetic electron traverse through the medium to interact further via transfer of energy to produce more charge carriers and/or secondary photons which then undergo elastic scattering or photoelectric absorption with the Bi_2_S_3_ nanoflowers. The secondary interactions also may produce more e-h pairs, in turn, adding to the overall charge carriers generated due to the incident beam. An external bias voltage establishes an electric field within the film of nanoflowers such that the radiation-induced e-h pairs can be drifted and collected at the electrodes and subsequently measured as electric current by the picoammeter.

Under an operating voltage of +1 V, the average photocurrent produced in the nanoflowers at 20, 23, 26 and 30 kV was measured to be 2.53, 3.1, 3.68, and 4.45 nA respectively. The photoresponse of the nanoflowers indicate an energy dependent behavior. The measurements were also compared to those recorded from a micro-ionization chamber. The ionization chamber measurements, listed in Table 1, confirmed similar trend of increase in the overall dose delivered (in terms of electrometer reading) when the x-ray tube-potential was increased. Evidently, the photocurrent measured from the Bi_2_S_3_ nanoflowers increased with the overall dose delivered at each of the tube potentials (20 to 30 kV). Detailed dose-dependent characteristics of the nanoflowers are presented in the next section.

### Dose Dependence

The dose was varied according to the inverse square law by changing the focus-to-surface distance (FSD). The dose was reduced in steps of 1/4th the initial value by increasing the FSD such that the dose of 1, 3/4, 1/2 and 1/4 times of the initial value, denoted as D-1, D-3/4, D-1/2, and D-1/4 respectively, were obtained for each of the x-ray energies. The measurements at each of the four different doses were compared to those recorded using the micro-ionization chamber. The fabricated devices and the micro-ionization chamber were exposed to x-rays for 18 s. The overall x-ray dose has been considered in terms of the electrometer readout displayed in units of cumulative charge (in nano-coulomb) as detected by the micro-ionization chamber. Figure 3 shows the x-ray induced photocurrents in Bi_2_S_3_ nanoflowers as a function of dose (electrometer readout indicated in the figure legends) for each of the four energies. Figure 4 show the same for the reference device. For all measurements, the devices were operated at +1 V external bias voltage. The nanoflowers showed relatively high (about eightfold signal amplification at maximum dose under 30 kV x-rays) and a constant photoresponse to changes in delivered dose when compared to the reference device (Figures 3 and 4).

### Field Size Dependence

Cumulative photocurrent from the Bi_2_S_3_ nanoflowers and the substrate was used to evaluate the effects of field sizes (exposure area) smaller than the active detection area (i.e. IDE coverage of 1 cm^2^). Figure 5 shows the cumulative photoresponse of the nanoflowers in comparison to those of the substrate as a function of field size for 20 and 30 kV x-rays. The incident field was varied by increasing the exposed area on the devices. Circular field sizes of 0.4, 0.6, 0.8, and 1 cm diameter, were determined with circular cut-out of lead sheet placed on top of the IDE with about 4 mm thick aluminum spacers over each side of the device so that the lead sheet was not directly in contact with the test device. The results were found to be in alignment with the features of an ideal dosimeter such that the radiation-induced currents in the nanoflowers were directly proportional to the exposed area (field-size) and several orders of magnitudes higher than the photocurrent of the substrate. Furthermore, it is evident that the Bi_2_S_3_ nanoflowers produced photocurrent for an area of exposure as small as 0.126 cm^2^ (i.e. for a field size determined by 0.4 cm diameter lead cut-out) at an x-ray tube-potential as low as 20 kV.

### Repeatability of measurements and Dependence on Bias Voltage

Measurement repeatability and its dependence on the bias voltage were studied for both the Bi_2_S_3_ nanoflowers and the substrate (without the nanoflowers) at each of the four x-ray tube potentials. Three different bias voltages were tested: +0.2, +0.4 and +1 V. The devices were exposed for an interval of 18 s followed by a longer interval of 1 min in order to assess the repeatability and the stability of the photoresponse of the Bi_2_S_3_ nanoflowers and the substrate (Figures 6 and 7). For each of the bias voltages, the photocurrent of the device with Bi_2_S_3_ nanoflowers was much higher than that of the reference device. Moreover, for all the measurements the magnitude of the “negative currents” at ‘x-ray-OFF state’ was found to be directly proportional to the applied voltage. In other words, charge trap and release mechanism occurred in the substrate (Au/Cr/SiNx/Si) resulting in storage of charge-carriers generated during irradiation followed by a discharge cycle (or reverse flow of charge-carriers) at the instance of ‘x-ray OFF’ state.

### Sensitivity

In order to evaluate the sensitivity of the Bi_2_S_3_ nanoflowers with respect to changes in delivered dose, the time-averaged photoresponse of the nanoflowers was plotted as a function of relative dose (D-1/4, D-1/2, D-3/4, and D-1 normalized to D-1 respectively where D-1 is indicative of maximum dose delivered because of the least distance of the x-ray source from the sample). Figure 8 shows a fairly linear relationship between the photocurrents from the nanoflowers and the relative dose delivered. The photoresponse of the nanoflowers increased by about 241%, for both 20 and 30 kV, when the dose was increased by four times the minimum value (i.e. maximum FSD of 30.4 cm from the sample). The signal from the Bi_2_S_3_ nanoflowers shows a dose-dependent behaviour i.e. the photocurrent increased linearly with increase in the x-ray dose (Figure 8). The overall photosensitivity of the Bi_2_S_3_ nanoflowers had a trend similar to the measurements obtained from the micro-ionization chamber. The photocurrent at 30 kV for the maximum dose was found to vary the most, with standard deviation of 91 pA, compared to those at other doses and energies.

Three main factors that affect sensitivity of a photoconductor are: (i) amount of radiation attenuated within the material, (ii) generation of charge carriers (i.e. e-h pairs), and (iii) charge collection efficiency^34^. Attenuation of the incident x-rays can be quantified in terms of the quantum efficiency (QE) which is given by the following equation:
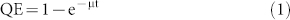
where μ is the linear attenuation coefficient and t is the thickness of the photoconductor. The attenuation coefficient is a function of the incident photon energy, Z, and density of the material. The mass attenuation coefficients for the energies of interest (determined from SpekCalc simulations) were calculated using WinXCom software (Table 2)^35^. The attenuation coefficient of the Bi_2_S_3_ nanoflowers increased with increase in the energy (tube potentials 20 to 30 kV) indicative of larger interaction cross-section for the incident photons at 20 kV compared to that at 30 kV. While it is favorable to have higher attenuation coefficient for more interaction, another factor to be considered is the penetration depth, defined as the reciprocal of linear attenuation coefficient. The penetration depth needs to be much lower than the thickness of the photoconductor for sufficient interaction (i.e. attenuation) with the incident x-ray photons. The penetration depth for each of the energies is also presented in Table 2.

Although large interaction cross-sections increase the possibility of charge carrier generation through ionization and/or excitation processes, the second factor that determines the sensitivity of the nanoflowers to mammographic x-rays is the ability to generate as many collectable charge carriers (e-h pairs) as possible per unit of absorbed radiation. The energy required for a single e-h pair generation, also known as the ionization energy (IE), of the nanoflowers can be roughly estimated from the bandgap energy (E_g_) by using the Klein rule for crystalline semiconductors: IE ≈ 3E_g_ = 3 × 1.33 eV = 3.99 eV^34^. The bandgap of the Bi_2_S_3_ nanoflowers (E_g_ = 1.33 eV) was obtained from the UV-Vis reflectance measurements. The narrow bandgap of 1.33 eV approximates to a relatively low IE of 3.99 eV for the nanoflowers. In other words, lower value of IE for photoconductor is favored to generate as many e-h pairs as possible upon irradiation thereby allowing higher sensitivity to x-rays. Finally, the last factor that affects the sensitivity is the charge collection efficiency (CCE) of the device. The CCE is directly proportional to the product of the charge carrier (e-h pair generated upon irradiation) drift mobility, its lifetime and the electric field applied across the electrodes (i.e. external bias voltage), and inversely proportional to the thickness of the photoconductor. In order to maximize CCE, there should be no loss of the charge carriers through recombination or trapping.

Since the Bi_2_S_3_ nanoflowers were dropcasted on the electrodes, the resultant film was apparently non-uniform in thickness. The reduction in the CCE and the variation in penetration depths for each of the energies have been considered to speculate the gradual loss in sensitivity observed for the repeatability measurements described earlier (Figure 6). Of all the energies and bias voltages, only 30 kV at an operating voltage of +1 V was found to have a relatively higher loss in the overall sensitivity for repetitive measurements. From the relatively high photoresponse measured at 30 kV, it is evident that the film of Bi_2_S_3_ nanoflowers has sufficient penetration depth (at least in some areas of the film) to produce maximum charge carriers (or photocurrent) at the maximum tube potential used in this study. Because of the uneven thickness of the Bi_2_S_3_ nanoflower film, the higher photoresponse at 30 kV (i.e. generation of more charge carriers) imply a higher possibility of charge trapping or recombination at this energy when compared to rest of the energies considered in this study. Furthermore, an overall temporal loss in the signal at the ‘x-ray ON’ states for 30 kV could also be attributed to possible reduction in the CCE. The CCE can be adversely affected from localized changes in sensitivity due to previous exposures (i.e. x-ray induced trap centers) and/or recombination of drifting charge-carriers with previously trapped oppositely charged carriers. The overall loss in sensitivity for 30 kV, is thus, speculated to be from the loss of charge carriers (reduction in the mobility × lifetime product) within the relatively thinner parts of the nanoflower film which possibly act as recombination/trapping sites.

It is interesting to note the effects of applied electric field on the sensitivity of the nanoflowers for all x-ray energies (tube potentials 20 to 30 kV). When the bias voltage across the IDE was increased from +0.2 to +1 V, the signal loss within each of the ‘x-ray ON’ states improved such that the photoresponse at +1 V for each ‘x-ray ON’ state was observed to be fairly stable in contrast to that at lower bias voltages. The higher electric field led to efficient charge separation and collection avoiding charge loss through bulk recombination thereby improving the CCE, and hence the sensitivity.

## Discussion

Hydrothermally synthesized nanoflower-like structures of Bi_2_S_3_ were investigated as a potential candidate for semiconductor-based x-ray sensing material. Recently, nanocrystalline materials have been reported to have interesting properties such as enhanced radiation resistance owing to the large volume fraction of grain boundaries that may serve as effective sinks for defects generated upon irradiation^24^. Reliability and durability are among the important features of an ideal dosimeter. Hence, the ‘self-repairing’ mechanism of nanomaterials may be exploited by extending their application in the development of novel, nanomaterial-based x-ray dosimeters with increased lifetimes. Moreover, the effective atomic number (Z_eff_) plays an important role in the interaction mechanisms between the x-rays and the target material. These interactions are directly responsible for the generation of charge carriers required for effective photoconductivity. The relatively high Z_eff_ of the Bi_2_S_3_ nanoflowers favors photoelectric interaction pathway even at the low x-ray energies and doses considered in this study. Photoresponse of commercial Bi_2_S_3_ powder with average unit size similar to that of the nanoflowers was briefly evaluated (Fig. S8 to S11 in the supplementary information file). In comparison, the Bi_2_S_3_ nanoflowers showed higher sensitivity and stability than the commercial Bi_2_S_3_ in powder form.

Instantaneous photoresponse of Bi_2_S_3_ nanoflowers to changes in x-ray energy/dose is evident from the results shown in Figures 2 and 3. Evaluation of different exposure areas, particularly those much smaller than the active region of detection, showed cumulative photocurrent in the order of several tens of nA at x-ray tube potential as low as 20 kV (Figure 5). The results indicate the possibility of using the Bi_2_S_3_ nanoflowers in miniaturized dosimetric applications. Furthermore, photoresponse of the Bi_2_S_3_ nanoflowers were found to be repeatable and stable for both short (18 s) and long (1 min) exposures (Figure 6). Except for the charge trap/release effects (“negative current” at the instance of ‘x-ray OFF’ state), the overall response of the substrate to x-rays was found to be negligible in comparison to that measured from the Bi_2_S_3_ nanoflower device (Figures 2 to 7). It is important to note that the overall sensitivity of the Bi_2_S_3_ nanoflower device showed similar trend to that of a micro-ionization chamber (Figure 8) at a minimal operating voltage of +1 V compared to the +300 V required for operating the ionization chamber.

To conclude, the performance of the Bi_2_S_3_ nanoflowers have been assessed under various conditions such as tube potentials and dose delivered in the mammographic range, bias voltages, and x-ray field sizes; all measurements were carried out under ambient conditions. The photoresponse of the nanoflowers clearly showed high sensitivity to changes in x-ray intensities, the capability to operate at a bias voltage as low as +0.2 V, and the potential to perform as a reliable dosimetric material for instantaneous dose measurements under mammographic x-rays.

## Methods

### Synthesis of Bi_2_S_3_ Nanoflowers

Bismuth nitrate pentahydrate (Bi(NO_3_)_3_.5H_2_O from Sigma Aldrich), thiourea and polyethylene glycol (PEG-4000) were used as precursors for hydrothermal synthesis of Bi_2_S_3_ nanoflowers. 0.322 g of Bi(NO_3_)_3_.5H_2_O was added to 8 ml of deionized (DI) water and stirred well. 1.288 g of PEG-4000 was dissolved in 2 ml of DI water, and stirred into the above solution. Finally, 1.288 g of thiourea was added and stirred well to obtain a clear, orange colored solution. The solution was then transferred into a 20 mL Teflon-lined stainless steel autoclave and exposed to 180°C for 17.5 hours. Black precipitates, obtained upon hydrothermal reaction, were washed several times with DI water, and finally with absolute ethanol, and dried overnight at 60°C in an oven.

### Characterization of Bi_2_S_3_ Nanoflowers

The samples collected from the hydrothermal reaction were studied using field emission scanning electron microscope (Zeiss LEO 1550), energy dispersive spectrometer (EDS) from Oxford Instruments Microanalysis System INCA Energy 350), and powder X-ray diffraction (XRD, Bruker D8 Advance diffractometer using Cu Kα radiation). The bandgap of Bi_2_S_3_ nanoflowers was determined from the diffuse reflectance spectra measured using Shimadzu UV-2501PC UV-Vis-NIR spectrophotometer equipped with an integrating sphere accessory, and BaSO_4_ as reference scatter. The details of the characterization studies can be found in the supplementary information file.

### Device Fabrication

Photolithographic techniques were used to fabricate Au/Cr IDE device. Firstly, 200 and 20 nm thick layers of Au and Cr, respectively, were deposited on clean silicon nitride coated wafer using Intlvac e-beam evaporator. Photoresist, S1811 (Shipley), was spin-coated on the silicon wafer and baked at 110° C for 90 s. A Mylar mask with 16 IDE patterns was used in this study. Each IDE pattern spanned over an area of 1 cm^2^ with an electrode spacing and width of 50 microns, and four contact pads, each with an area of 2 mm^2^. The patterns were transferred from the mask onto the photoresist-coated wafer by flood exposure to ultraviolet radiation using Suss MA6 Mask Aligner. The photoresist layer was then developed in MF-319 (Shipley's photoresist developer); the wafer was, subsequently, dipped in DI water and dried with nitrogen gas. Finally, the hydrothermally synthesized Bi_2_S_3_ nanoflowers were dispersed in ethanol (12.4 mg/200 μL) of which 15 μL was carefully pipetted on the 1 cm^2^ area of the IDE pattern using the dropcast method. The film was dried at room temperature. Electrical connections were cold soldered on a pair of contact pads using conductive silver epoxy. Since Bi_2_S_3_ nanoflowers were found to be photosensitive in the UV-Vis range, the devices were stored in the dark.

### Experiment Setup

The photoresponse of the Bi_2_S_3_ nanoflowers on Au/Cr IDE was measured using mammographic x-ray energies with tube potentials: 20, 23, 26 and 30 kV. The experiments were conducted using a superficial x-ray facility (Gulmay Medical Inc.) at the Grand River Regional Cancer Center (Kitchener, Ontario, Canada). An aperture of diameter 1 cm was used for all measurements. The x-ray tube-current was set to 20 mA for all tube potentials. No external filters were used.

### Measurements

Both dark (leakage) current and x-ray induced currents from the Bi_2_S_3_ nanoflowers and the substrate (the Au/Cr/SiNx/Si IDE without the nanomaterial) were measured for the following conditions: (i) Four tube potentials in the mammographic range of 20, 23, 26, and 30 kV. (ii) Four different doses were delivered by varying the FSD for each of the four tube potentials. (iii) Three different bias voltages: +0.2, +0.4 and +1 V for each of the four tube potentials. (iv) Four different field sizes (0.4, 0.6, 0.8 and 1 cm) for two peak voltages (20 and 30 kV). A picoammeter (Keithley 6487A) was used to record all measurements. The dark current for both devices (with and without Bi_2_S_3_ nanoflowers), for a given bias voltage, was accounted for through subtraction from the picoammeter measurements. All measurements were carried out under ambient conditions but in the dark since Bi_2_S_3_ nanoflowers were found to be photosensitive in the UV-Vis range. Micro-ionization chamber (Exradin 0.016 cc, model A14) along with an electrometer (Dose-1) was used to measure the dose (i.e. cumulative charge over the exposure period) for all energies.

### Simulations

SpekCalc software was used to simulate the x-ray emission spectra for each of the tube-potentials (20, 23, 26 and 30 kV) used in this study. The mean energy estimated from SpekCalc for each tube-potential was considered as a monoenergetic beam and used to calculate energy-weighted effective Z of Bi_2_S_3_ nanoflowers using the Auto-Zeff simulation software. The mass attenuation coefficient (in g/cm^2^) of the nanoflowers for each of the mean energies (calculated using SpekCalc for all four tube-potentials) was obtained from WinXCom simulation tool. The corresponding linear attenuation coefficients (in cm^−1^) were calculated by multiplying the density of the Bi_2_S_3_ nanoflowers with the mass attenuation coefficients. Finally, the penetration depth of the incident photons for each of the tube-potentials was calculated from the reciprocal of the linear attenuation coefficient. The screenshots of the simulations are provided in the supplementary information file.

## Author Contributions

S.N. synthesized the Bi_2_S_3_ nanomaterial and performed the experiments. S.N., E.K.O. and J.T.W.Y. analyzed the data. S.N. wrote the manuscript based on inputs from E.K.O. and J.T.W.Y. All authors reviewed the manuscript.

## Supplementary Material

Supplementary Information Supplementary Information File

## Figures and Tables

**Table 1:** Electrometer measurements using micro-ionization chamber for an exposure time of 18 s for all tube potentials

Tube Voltage (kV)	20	23	26	30
Electrometer readout for cumulative charges (nC)	2.12	2.87	3.64	4.72

**Table 2:** Mass attenuation coefficient and the corresponding penetration depth for Bi_2_S_3_ nanoflowers at each of the tube potentials as determined from SpekCalc simulations and calculated using WinXCom software^35^

X-ray tube potential (kV)	Mean energy (keV)	Mass attenuation coefficient (cm^2^/g)	Penetration depth (μm)
20	9.78	127	11.6
23	10.6	103	14.32
26	11.4	85.9	17.17
30	12.4	69.3	21.28
