# Supplementary material for: Bismuth Sulfide Nanoflowers for Detection of X-rays in the Mammographic Energy Range
Source: Sci Rep. 2015 Mar 24;5:9440. doi: 10.1038/srep09440 (PMC4371100; doi:10.1038/srep09440)
Supplement: Supplementary Information — File [file srep09440-s1.pdf]

Supplementary Information

Bismuth Sulfide Nanoflowers for Detection of X-rays in the Mammographic Energy Range

Shruti Nambiar[a,b], Ernest K. Osei[a,c,d], John T.W.Yeow[*,a,b]

[a]Department of Systems Design Engineering, 200 University Avenue West, University of Waterloo, Waterloo, Ontario, Canada, N2L 3G1.

[b]Waterloo Institute for Nanotechnology, 200 University Avenue West, University of Waterloo, Waterloo, Ontario, Canada, N2L 3G1.

[c]Department of Medical Physics, Grand River Regional Cancer Centre, Kitchener, Ontario, Canada, N2L 3G1.

[d]Department of Physics and Astronomy, 200 University Avenue West, University of Waterloo, Waterloo, Ontario, Canada, N2L 3G1.

Table of Contents

Energy dispersive spectrometry S-1
Powder x-ray diffraction analysis S-2
SpekCalc simulation S-3
WinXCom simulation S-4
Auto-Zeff simulation S-5
Energy bandgap calculation S-5
Photoresponse of commercial Bi_2S_3 powder S-7
References S-11

Energy dispersive spectrometry

The chemical composition of the nanoflowers was confirmed with energy dispersive spectrometer (Oxford Instruments Microanalysis System INCA Energy 350) as part of the scanning electron microscope (SEM, JOEL JSM-6460). The sample was dispersed in ethanol and dropcasted on aluminum foil attached to a silicon substrate. Results of the elemental analysis are presented in this section (Figures S1, S2, and Table S1).

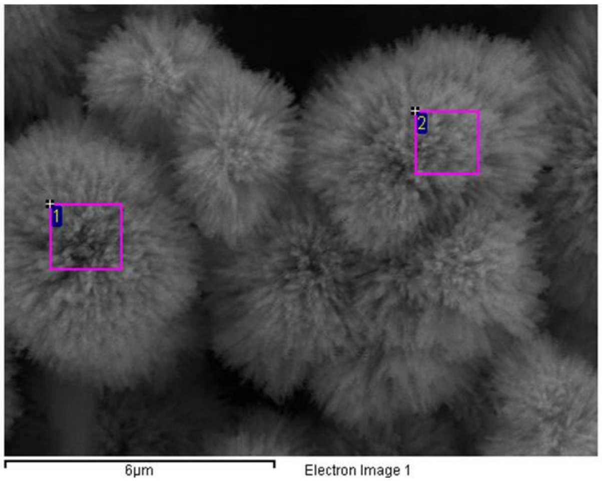

Figure S1: SEM image of the Bi_2S_3 nanoflowers with two regions (shown as 1 and 2) selected for composition analysis.

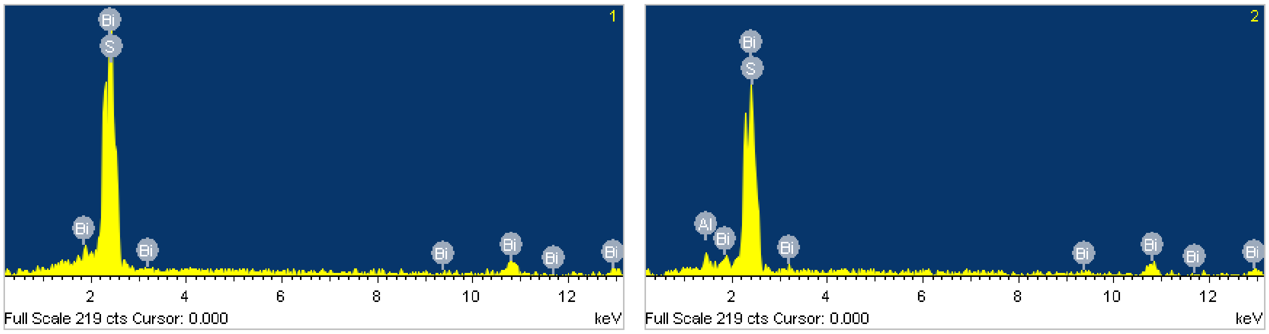

Figure S2: Energy dispersive spectra showing peaks for (1) Bi and S, and (2) Al (substrate), Bi, and S

Table S1: Quantitative analysis of regions 1 and 2

Spectrum	Al	S	Bi	Total
1		18.77	81.23	100.00
2	1.90	18.11	79.99	100.00
Max.	1.90	18.77	81.23	
Min.	1.90	18.11	79.99	

All results in weight%

Powder x-ray diffraction analysis

Figure S3 shows the powder x-ray diffraction (XRD) pattern of the nanoflowers recorded on a Bruker D8 Advanced X-ray diffractometer using the parameters listed in Table S2. The XRD pattern shows polycrystalline nature and all the peaks in the XRD pattern can be indexed to orthorhombic Bi_2S_3 (JCPDS 17-0320) with no indication of impurities (Figure S3).

Table S2: Parameters used in XRD study

Radiation source	Cu Kα
diffraction angle of 2θ	10–70°
Step size	0.019218° 2θ
Specimen Motion	15 rpm

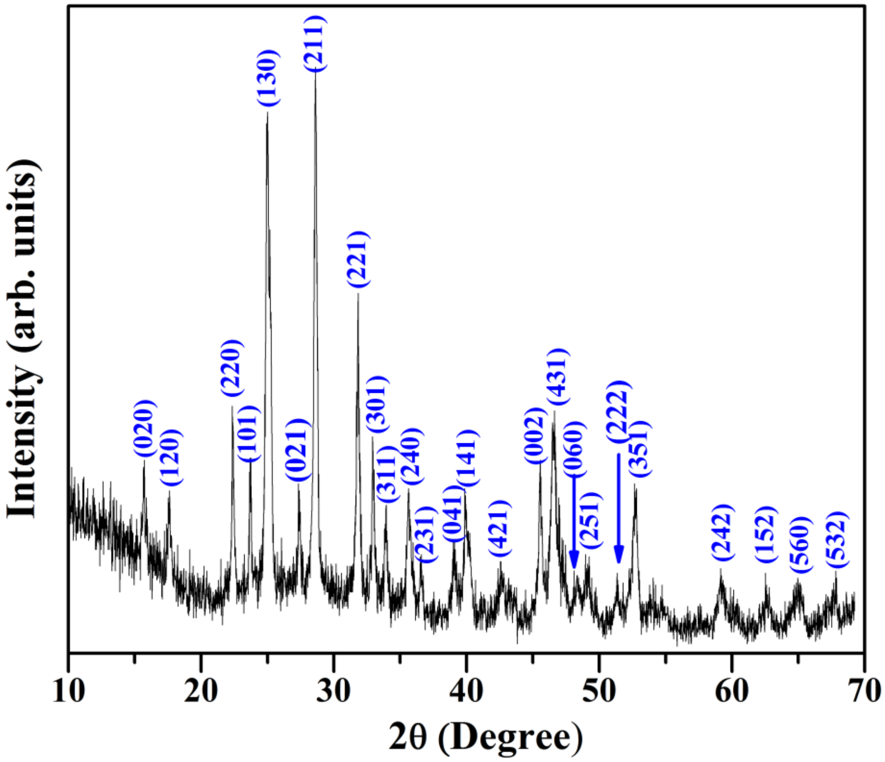

Figure S3: XRD pattern of the nanoflowers

SpekCalc simulation

Figure S4 shows the spectra for x-ray tube-potentials 20, 23, 26 and 30 kV using the SpekCalc simulation software. The "air thickness" parameter of 154 mm was used to account for the FSD of 150 mm (from the x-ray setup), and an additional 4 mm from the distance between the tip of the 1 cm cone and the surface of the test device. The mean x-ray energy for each of the tube-potential was also obtained from the simulations.

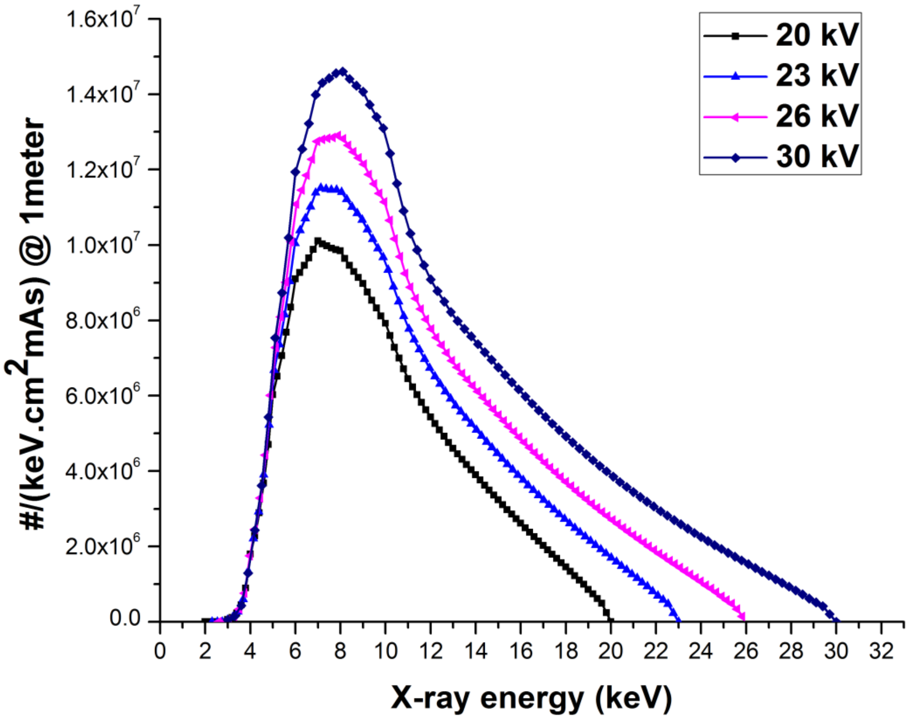

Figure S4: X-ray spectrum for tube potentials – 20, 23, 26, and 30 kV

WinXCom simulation

The mass attenuation coefficients (cm^2/g) of Bi_2S_3 for the mean x-ray energies: 9.78, 10.6, 11.4, and 12.4 keV (output from SpekCalc simulations) were obtained using the WinXCom program as shown in Figure S5.

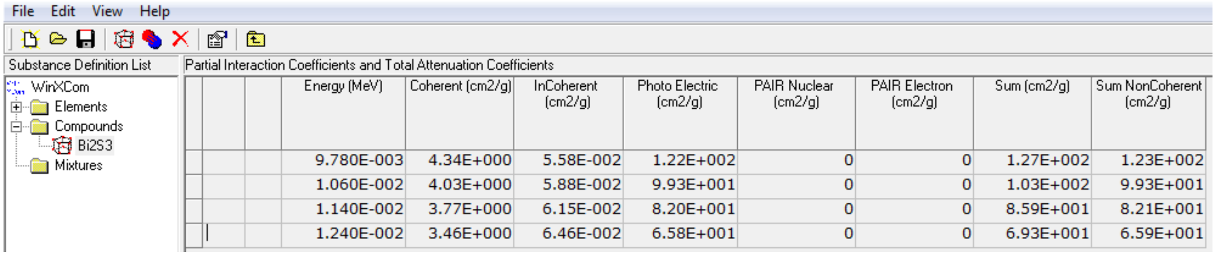

Energy (MeV)	Coherent (cm2/g)	InCoherent (cm2/g)	Photo Electric (cm2/g)	PAIR Nuclear (cm2/g)	PAIR Electron (cm2/g)	Sum (cm2/g)	Sum NonCoherent (cm2/g)
9.780E-003	4.34E+000	5.58E-002	1.22E+002	0	0	1.27E+002	1.23E+002
1.060E-002	4.03E+000	5.88E-002	9.93E+001	0	0	1.03E+002	9.93E+001
1.140E-002	3.77E+000	6.15E-002	8.20E+001	0	0	8.59E+001	8.21E+001
1.240E-002	3.46E+000	6.46E-002	6.58E+001	0	0	6.93E+001	6.59E+001

Figure S5: X-ray interaction cross-sections for each of the input energies as displayed in WinXCom software.

Auto-Zeff simulation

Energy-weighted effective atomic number (Z_{eff}) of Bi_2S_3 nanoflowers was estimated using Auto-Zeff software (Figure S6). The Z_{eff} was found to be in the range of 45 to 46.5 for the x-ray energies used in this study (9.78, 10.6, 11.4, and 12.4 keV).

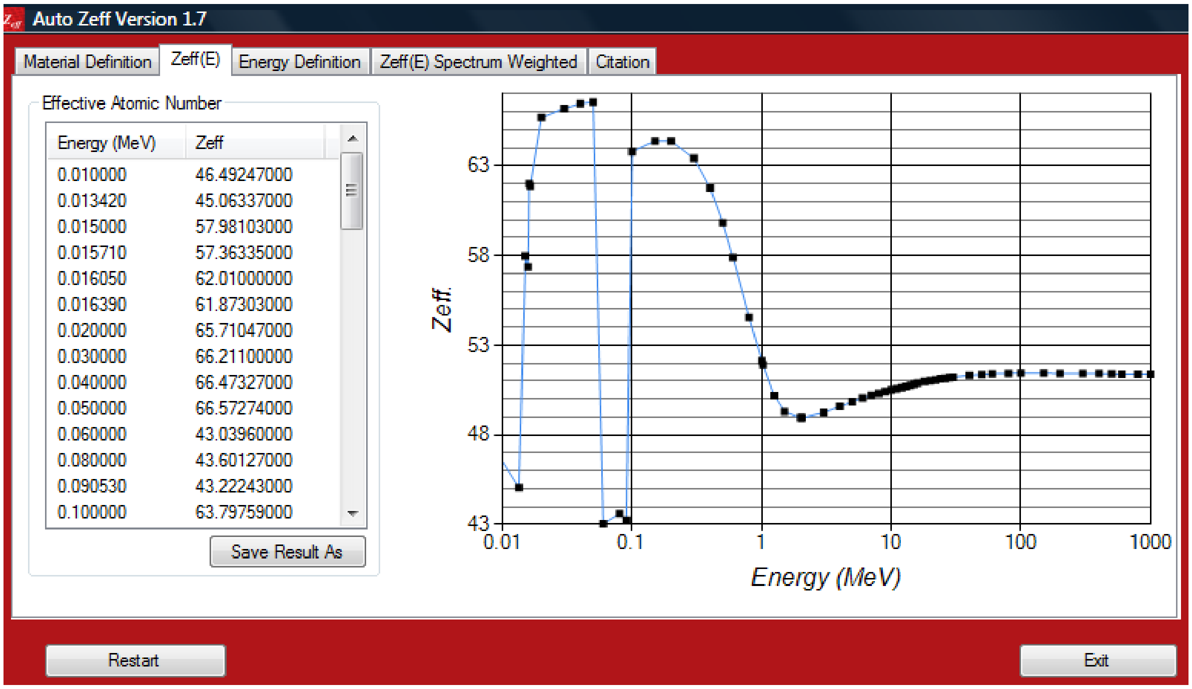

Figure S6: Energy-weighted Z_{eff} of Bi_2S_3 for x-ray energies from 10 to 100 keV as simulated in Auto-Zeff program.

Energy bandgap calculation

The bandgap of Bi_2S_3 nanoflowers was calculated from the diffuse reflectance spectra (%R versus wavelength) measured using UV-Vis-NIR spectrophotometer (Shimadzu UV-2501PC). The absorbance (F(R)) was calculated from the diffuse reflectance spectrum using the Kubelka-Munk function[1], and the energy bandgap (E_g) was then estimated by substituting F(R) in the Tauc equation.

Kubelka-Munk function is given by:

$$F(R) = \frac{\left(1 - \left(\frac{R}{100}\right)^2\right)}{\left(1 - 2\left(\frac{R}{100}\right)\right)} \qquad (1)$$

where, R is the measured diffuse reflectance.

For the UV-Vis-NIR range, the optical absorption coefficient (α) can be determined by the Tauc equation:

$$\alpha = A\frac{(\mathrm{h\nu} - \mathrm{Eg})^{\mathrm{n}}}{\mathrm{h\nu}} \qquad (2)$$

where, hν is the photon energy, E_g is the bandgap, and A and n are constants.[2] Since Bi_2S_3 is a direct bandgap material, n=1/2. By substituting α with F(R) in equation (2), the Tauc plot ($(F(R) * h\nu)^2$ vs. hν) was obtained and the energy bandgap (E_g) was then estimated by extrapolating the linear portion of the plot to the energy axis as shown in Figure S7. The bandgap was found to be 1.33 eV.

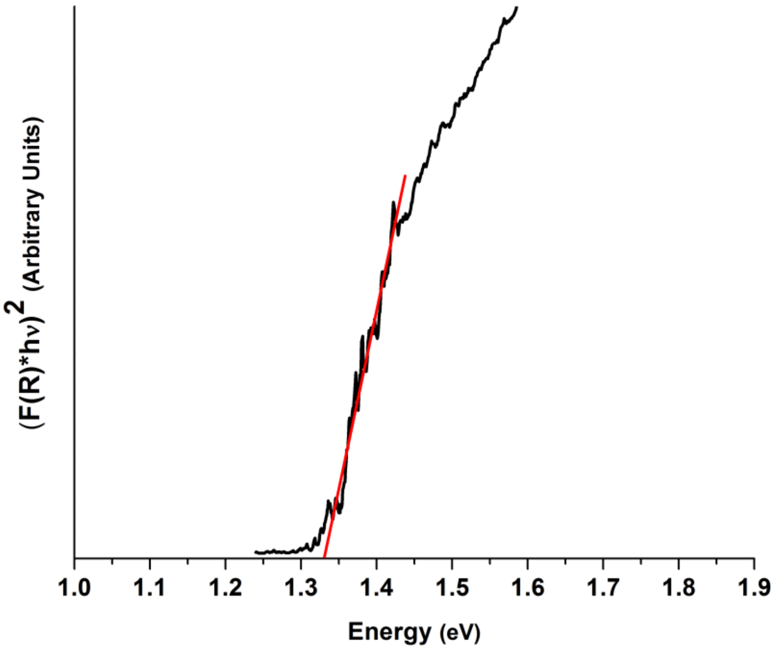

Figure S7: Tauc plot for Bi_2S_3 nanoflowers

Photoresponse of commercial Bi_2S_3 powder

For comparative analysis of the photoresponse of the Bi_2S_3 nanoflowers with those of the commercial product, a device with a film of Bi_2S_3 powder (MKN-Bi2S3-900 from M. K. Impex Co., MKnano Division) was fabricated using the technique employed for the device with the Bi_2S_3 nanoflowers. The morphology and unit size of the as-purchased Bi_2S_3 powder was determined using FESEM (Figure S8). The powder consisted of irregularly shaped particulates of Bi_2S_3 with an average unit size of 5 microns, which is comparable to the unit size of the Bi_2S_3 nanoflowers. The dark current measured from the commercial powder was >2.5 times the dark current observed in the nanoflower device.

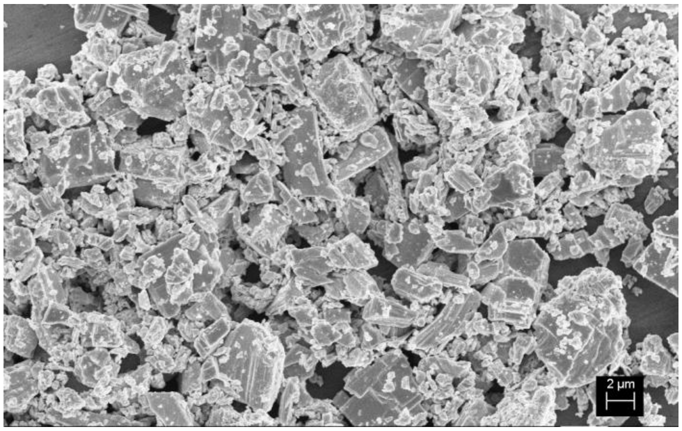

Figure S8: FESEM image of commercial Bi_2S_3 powder

The photoresponse of the device, operated under +1V bias voltage, upon exposure to different x-ray tube potentials (20 to 30 kV) for an interval of 1 min is shown in Figure S9. A rapid loss of charge carriers followed by a plateau after ~50 s can be observed. The average photoresponse of the fairly steady portion of the curve (between 50 to 60 s time interval) at 20, 23, 26 and 30 kV was found to be 1.46, 1.85, 2.31, and 2.83 nA respectively. In comparison, the average photocurrent produced in the nanoflowers at 20, 23, 26 and 30 kV was measured to be 2.53, 3.1,

3.68, and 4.45 nA respectively. Moreover, the photoresponse curves of the nanoflowers showed significantly higher stability in comparison to the commercial product with irregular unit structures. Repeatability and effects of different bias voltages were also evaluated under 30 kV x-rays. The rapid loss of photocurrent was evident for all exposures, however, the overall photoresponse was fairly repeatable for both short- and long-term exposures (Figure S10).

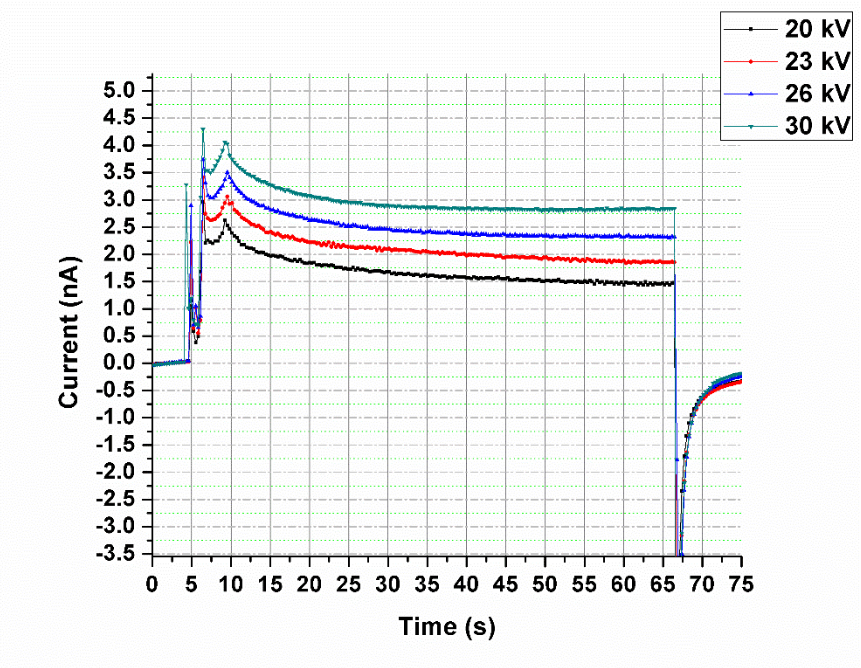

Figure S9: Photoresponse of the device with commercial Bi_2S_3 powder exposed to different x-ray tube potentials 20 to 30 kV.

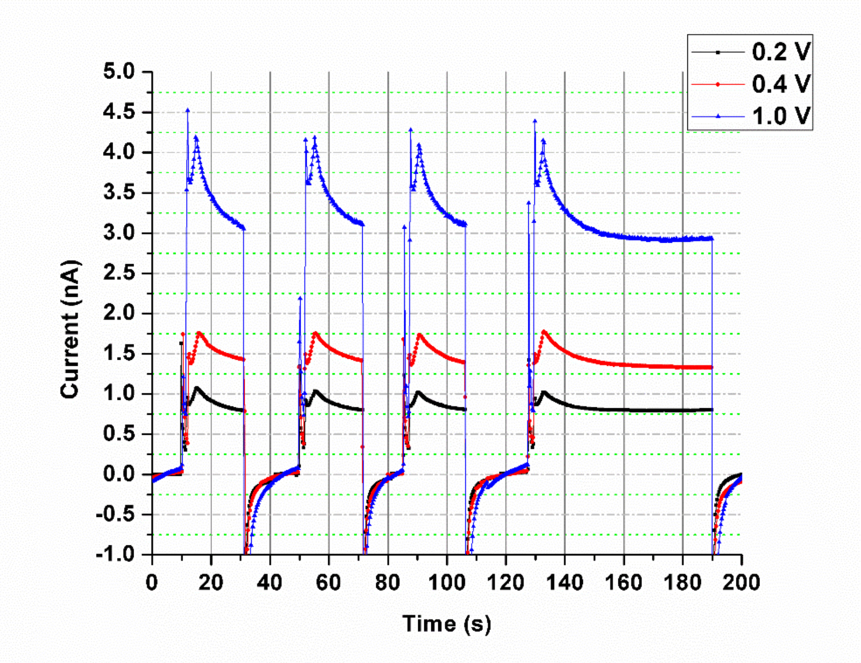

Figure S10: Repeatability assessment for short- (18 s) and long-term (1 min) exposures to 30 kV x-rays with the device operated under three different bias voltages indicated in the legend.

Dose dependence and sensitivity of the commercial Bi_2S_3 powder was also evaluated under 20 and 30 kV x-rays (Figure S11). As expected, commercial Bi_2S_3 was also found to be appreciably sensitive to dose changes at the tube potentials as low as 20 and 30 kV. However, in addition to the rapid loss in x-ray induced signal over time, the overall sensitivity (percentage change in photocurrent at maximum dose with respect to the minimum) was found to be relatively lower at both at 20 kV (168.5% for the powder and 241% for the nanoflowers) and 30 kV (217% for the powder and 241% for the nanoflowers) in comparison with the nanoflowers at the same tube potentials and doses.

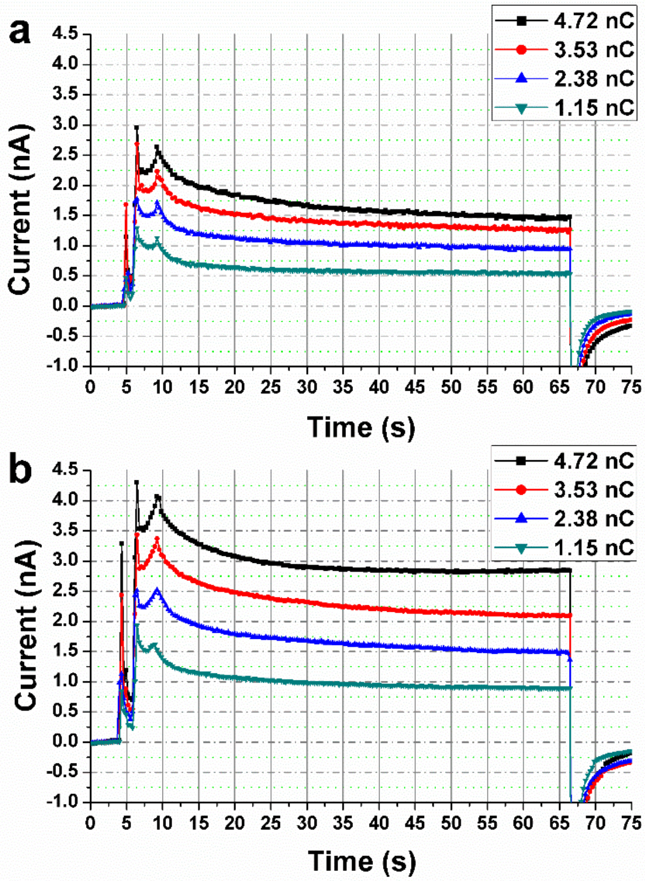

Figure S11: Photoresponse of the commercial Bi_2S_3 powder to four different x-ray doses at a tube potential of (a) 20 kV, and (b) 30 kV. For each tube potential, the electrometer readout (measurements from the micro-ionization chamber) corresponding to the four different x-ray doses are shown in the legend. The exposure time was set to 1 min for all measurements.

In summary, the device with Bi_2S_3 nanoflowers showed better overall performance in comparison with the device with the commercial powder. The differences between the results may be due to several factors that may include the synthesis process used for the commercial powder, which may have affected the crystallinity of the material (critical for charge transport)

and /or introduced defect or trap states (resulting in charge recombination/trapping), and finally, the role of nanoscale structures in photoelectric absorption of x-rays. During photoelectric interaction, the photon energy may be transferred to the target atom in the form of Auger and delta electrons, photoelectrons, or characteristic x-rays. Auger and delta electrons have very short range (less than 1 micron), photoelectrons can go up to hundreds of microns and the characteristic x-rays can go as far as centimeters.[3] In order to consider the possible role of nanostructure in the context of x-ray detection, one may speculate that high atomic number, 3D nanocrystalline material, such as Bi_2S_3 nanoflowers containing numerous 1D nanorods, may generate more collectible charge-carriers (owing to the short range of the Auger and delta electrons) and subsequently, provide enhanced photoresponse in comparison to 1D or 2D nanostructures or bulk (powder) of Bi_2S_3. However, detailed experiments and simulations need to be performed in order to have a clear understanding of the factors leading to the differences in the device performances.

References

1 Kubelka, P. & Munk, F. Ein Beitrag zur Optik der Farbanstriche. *Zeits. f. Techn. Physik* **12**, 593–601 (1931).

2 Mane, R. S., Sankapal, B. R. & Lokhande, C. D. Studies on chemically deposited nanocrystalline Bi_2S_3 thin films. *Mater. Res. Bull.* **35**, 587-601 (2000).

3 Lechtman, E. *et al.* Implications on clinical scenario of gold nanoparticle radiosensitization in regards to photon energy, nanoparticle size, concentration and location. *Phys Med Biol* **56**, 4631-4647 (2011).
